# The direct Myc target Pim3 cooperates with other Pim kinases in supporting viability of Myc-induced B-cell lymphomas

**DOI:** 10.18632/oncotarget.283

**Published:** 2011-06-05

**Authors:** Linus Plym Forshell, Yongmei Li, Tacha Zi Plym Forshell, Martina Rudelius, Lisa Nilsson, Ulrich Keller, Jonas Nilsson

**Affiliations:** ^1^ Department of Molecular Biology, Umeå University, Umeå, Sweden; ^2^ Department of Medical Microbiology, Tianjin Medical University, Tianjin, People's Republic of China; ^3^ Department of Pathology, Technical University of Munich, Munich, Germany; ^4^ Department of Medicine, Technical University of Munich, Munich, Germany

**Keywords:** cancer, lymphoma, oncogenes, c-Myc, Pim-3

## Abstract

The Pim kinases are weak oncogenes. However, when co-expressed with a strong oncogene, such as c-Myc, Pim kinases potentiate the oncogenic effect resulting in an acceleration of tumorigenesis. In this study we show that the least studied Pim kinase, Pim-3, is encoded by a gene directly regulated by c-Myc via binding to one of the conserved E-boxes within the *Pim3* gene. Accordingly, lymphomas arising in Myc-transgenic mice and Burkitt lymphoma cell lines exhibit elevated levels of Pim-3. Interestingly, inhibition of Pim kinases by a novel pan-Pim kinase inhibitor, Pimi, in Myc-induced lymphoma results in cell death that appears independent of caspases. The data indicate that Pim kinase inhibition could be a viable treatment strategy in certain human lymphomas that rely on Pim-3 kinase expression.

## INTRODUCTION

Tumorigenesis is a multistep process where several acquired genetic alterations, or hallmarks, occur leading to transformation of a cell [1]. c-Myc is a transcription factor with more than 1000 target genes (www.myccancergene.org), many of which directly impinge on the hallmarks of cancer. Therefore, Myc expression is often selected for during various steps in the genesis of many types of human cancer. To avoid transformation, normal cells can respond to Myc overexpression by undergoing apoptosis or senescence [2, 3]. Thus, for Myc overexpression to drive transformation additional genetic alterations affecting apoptotic signaling are required [4], thereby allowing the state of genomic instability that facilitates the required accumulation of mutations.

The Pim kinase family is a group of three serine/threonine kinases encoded by genes that were identified as hotspots for proviral integration of Moloney murine leukemia virus (Pim) in retrovirus-induced lymphomas [5]. The three kinases, Pim-1, Pim-2 and Pim-3, are considered weakly oncogenic when expressed as transgenes [6-8]. However, when co-expressed with Myc they strongly accelerate Myc-driven lymphomagenesis [6, 9-11]. The Pim kinase genes are located on different chromosomes but they encode proteins with similar sizes and with a high degree of homology [12].

The Pim kinases phosphorylate a wide variety of cellular targets involved in regulating the cell cycle, including cyclin-dependent kinase inhibitors p21 and p27 [13-15], cell cycle proteins such as CDC25A and CDC25C [16, 17]and suppressors of cytokine signaling SOCS1 and SOCS3 [18, 19]. Moreover Pim kinases also phosphorylate proteins involved in translation such as eIF4B [20] and 4EBP1 [21], pro-apoptotic Bcl-2 family member Bad [21-24] and transcriptional regulators such as Myc [25], Myb [26], RUNX1 and RUNX3 [27]. Recently it was shown that Pim-1 can bind the Myc-Max complex and so get recruited to E-boxes from where Pim-1 can phosphorylate serine 10 on histone H3 [28]. This phosphorylation regulates up to 20% of the Myc transcriptional targets, some of which are needed for Myc driven transformation. It is not known whether all of the above-described functions represent separable functions of the different Pim family members or if significant redundancies exist. Nevertheless, single knockout mice do not have any overt phenotype whereas triple *Pim1*^−/−^;*Pim2*^−/−^;*Pim3*^−/−^ knockout mice are smaller and exhibit a small defect in lymphomcyte stimulation capacity [12].

Despite the strong genetic links between Myc and Pim kinases emanating from experimental models of cancer, it is not clearly established whether or not activation of Pim kinases occurs spontaneously in Myc-induced lymphomas. To address this we analyzed B-cell tumors that arose in λ-*Myc* transgenic mice [29], which express c-Myc under the control of the immunoglobulin l enhancer. To our surprise we find that the most highly expressed Pim kinase in these lymphomas is Pim-3, and that *Pim3* is a Myc target gene. These data may partly explain why proviral activation of *Pim1* and *Pim2* in Eμ-*Myc* mice occurs most frequently – *Pim3* is already activated by Myc. Importantly, we also show that inhibition of Pim kinases induces cell death of Myc-induced lymphomas.

## RESULTS

### Pim-3 kinase protein levels are elevated in Myc over expressing tumors

**Figure 1:**
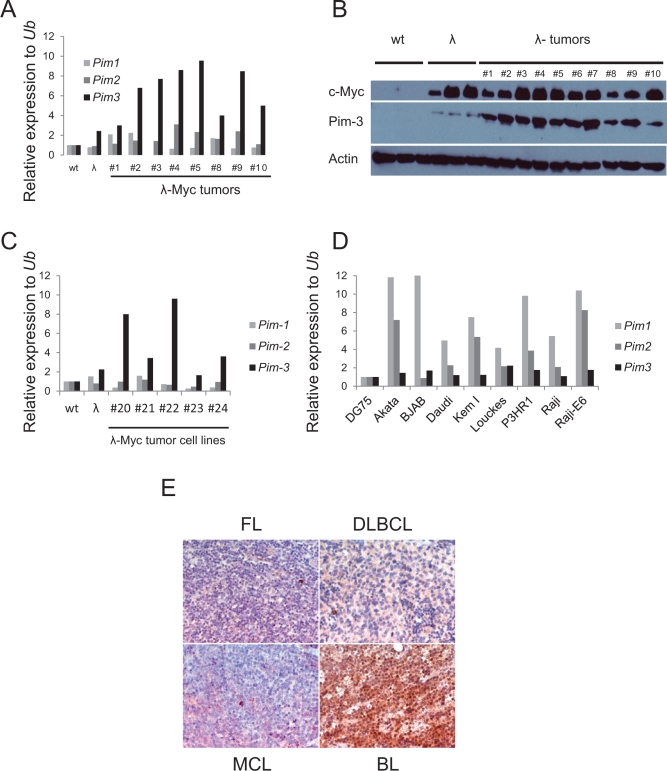
Pim-3 expression is elevated in mouse and human lymphoma tissue (A) Quantitative reverse transcription PCR (qRT-PCR) analysis of *Pim* kinase expression in B cells from wildtype and precancerous *λ-Myc* transgenic mice (λ), and in lymphomas harvested from *λ-Myc* transgenic mice. *Pim kinase* levels were normalized to that of ubiquitin (*Ub*) (B) Western blot analysis of Pim-3 kinase (using an antibody from Cell signaling) in same material as analyzed by qRT-PCR. (C) qRT-PCR analysis of *Pim*-kinase expression in cell lines established from *λ-Myc* and Eμ-*Myc* transgenic mice. (D) qRT-PCR analysis of *Pim*-kinase expression in Human Burkitt lymphoma cell lines. The expression levels are relative to that of one of the cell lines, DG75. (E) Immunohistochemistry analysis of Pim-3 expression in various lymphomas from patients. The antibody was from Abcam.

Although considered weak oncogenes, all Pim kinase family members have been shown to strongly potentiate Myc driven tumorigenesis as proviral insertion targets or transgenes [6, 9-11]. Although these landmark studies established a genetic link between Myc and Pim kinases, they did not address whether this synergy could be established spontaneously in a reciprocal manner. To partly address this we analyzed B-cell tumors from λ-*Myc* transgenic mice for Pim kinase mRNA expression levels. Quantitative reverse transcriptase PCR (qRT-PCR) showed that elevated RNA levels of *Pim1* and *Pim2* could be observed in some tumors but that *Pim3* was the only Pim kinase family member with significantly elevated mRNA levels in all tumors as compared to wildtype B cells (Figure 1A). Interestingly, elevated *Pim3* mRNA and Pim-3 protein were seen not only in tumors but also in B cells harvested from 4-6 week old precancerous mice with no signs of lymphoma (Figure 1A and 1 B). Moreover, tumor cell lines established from λ-*Myc* and Eμ-*Myc* transgenic mouse B cell tumors still exhibited elevated mRNA levels of *Pim3*, suggesting the importance of maintaining expression even in conditions of surplus growth factors (Figure 1C). To analyze which of the Pim kinases were expressed in human lymphomas, we analyzed a set of human Burkitt lymphoma (BL) cell lines by qRT-PCR. When Pim kinase expression in the human BL cell line DG75 was set to 1, it appeared as if *Pim1* and *Pim2* mRNA is elevated in all the BL cells (Figure 1D). However, when analyzing the Ct values it became obvious that *Pim3* was the more abundant Pim transcript because a signal was obtained at cycle 21, as opposed to cycle 23 or higher for *Pim1* and *Pim2* mRNAs. Moreover, immunohistochemistry analysis of Pim-3 expression in mantle cell lymphoma (MCL), follicular lymphoma (FL), diffuse large cell B-cell lymphoma (DLCBL) and BL showed that BL is the lymphoma-type that exhibits the highest Pim-3 expression (Figure 1E). Taken together, the data indicate that Pim-3 is the main Pim kinase overexpressed in Myc-induced lymphomas from mice and patients whereas Pim-1 and Pim-2 are more sporadically overexpressed.

### Pim3 is a direct Myc transcriptional target

**Figure 2:**
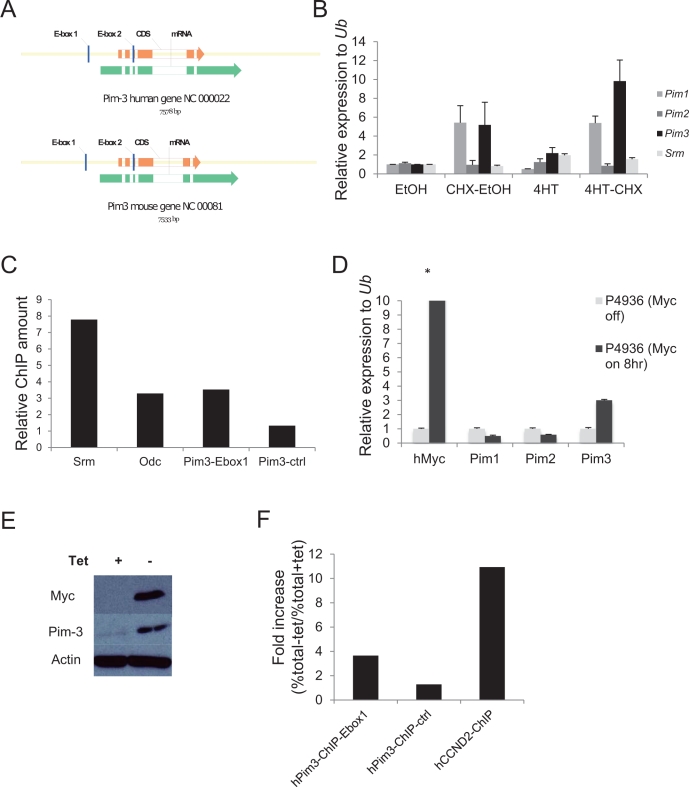
The Pim3 gene has evolutionary conserved E-boxes, which mediate Myc-regulated transcription (A) Schematic representation of mouse and human *Pim3* loci, which contain two evolutionarily conserved E-boxes (CACGTG). (B) NIH 3T3 fibroblasts carrying a MycER construct were treated with the estrogen receptor (ER) agonist 4-hydroxytamoxifen (4-HT) with or without the translation inhibitor cycloheximide (CHX) for 4 h and were then harvested for qRT-PCR analysis of *Pim* expression. *Srm*, a Myc target gene with five E-boxes, was used as a positive control. (C) Chromatin immunoprecipitation (ChIP) assay was run on λ820 cells using a Myc antibody to immunoprecipitate the E-box regions. Immunoprecipitated chromatin was subsequently analyzed by qPCR utilizing primers directed against E-box regions of the Myc target genes Srm and Odc but also against the E-box1 in the *Pim3* locus. As a negative control primers designed not to recognize the *Pim3* gene (Pim3-ctrl) were used. Percent total chromatin immunoprecipitated was calculated using the equation % total = 2 ^(Ct input - Ct ChIP)^ x % input and was presented as “Relative fold increase” calculated accordingly, % total sample / % total negative control. The negative control value was generated by immunoprecipitation using normal rabbit IgG. Shown is one of two independent experiments yielding similar results. (D) The human BL cell line P493-6 carrying a tetracycline (tet-off) regulatable system for Myc expression was cultured with 0.1μg/ml tetracycline (tet) or without tet for 8 hours, after which cells were harvested for qRT-PCR analysis of *Pim* and *Myc* transcripts (E) P493-6 cells from the same experiment were also harvested for western blot analysis of Pim-3 and Myc protein expression. (F) P493-6 cells were cultured with tetracycline for 72 hours and then divided into two sub-cultures, one with tetracycline (myc off) and one without (myc on), and were cultured for another 8 hours before being harvested for ChIP analysis. Chromatin was immunoprecipitated using a Myc antibody and the qPCR was run with primers directed against the *PIM3* E-box1, primers not recognizing the *PIM3* gene as negative controls, and primers against *CCND2* as a positive control. Percent total chromatin immunoprecipitated was calculated as described above. Data were presented as “Fold increase (%total – tet / %total+tet)”. Shown is one of two independent experiments yielding similar results.

Myc transcription is mediated through E box sequences, most often CACGTG [30-37]. To investigate if the high levels of Pim-3 in Myc-expressing cells and tumors were due to a direct induction of transcription, we first analyzed the nucleotide sequence of the *Pim3* locus and found that it contains two potential E-boxes, which were conserved in mice and man (Figure 2A). We then infected NIH3T3 fibroblasts with a retrovirus expressing Myc-ER, an inducible form of Myc that can be activated by adding the estrogen analog 4-hydroxytamoxifen (4-HT) to the culture medium. Addition of 4-HT, with or without the translation inhibitor cycloheximide, showed that the mRNA of the direct Myc target *Srm* could be induced 2-fold even when translation was inhibited, as could *Pim3* mRNA (Figure 2B). Because *Pim3* also was induced by cycloheximide, which complicates interpretation of these types of experiments, we analyzed whether Myc binds the E-boxes of the *Pim3* locus by performing chromatin immunoprecipitation assay (ChIP) on formaldehyde cross-linked DNA from a λ-*Myc* transgenic mouse B-cell lymphoma cell line, λ820, established in our laboratory [38]. Indeed, when ChIP was performed using primers designed to flank E-box 1 of *Pim3*, the signal obtained was comparable to that of the signal obtained with primers against *Srm* and *Odc* (Figure 2C), two confirmed Myc transcriptional targets [39, 40]. Primers against a sequence one kb downstream of the *Pim3* gene was used as negative control, and yielded signals 3-7 times weaker. To assess if Myc directly regulates *PIM3* in human cells we used the P493-6 lymphoma cell line, which has a tetracycline (Tet) regulatable Myc cassette, which is silent in the presence of tetracycline [41]. P493-6 cells were incubated 72 hours in the presence of tetracycline (Myc off) after which they were split in two cell culture flasks, one culture with tetracycline (Myc off) and one culture without tetracycline (Myc on). The two cultures were then incubated for 8 hours, after which cells were harvested for mRNA, protein and ChIP analysis. We ran a qRT-PCR on mRNA harvested from these samples to measure the *PIM* levels with and without Myc expression. In accordance with the mouse results expression of Pim-3 kinase, but not Pim-1 or Pim-2, was induced by Myc activation at the transcriptional (Figure 2D) and translational level (Figure 2E). Moreover, ChIP experiments performed using a Myc antibody showed that the *PIM3* E-box 1 was immunoprecipitated from the same P493-6 cell preparation, although to a lesser extent than the Myc target gene Cyclin D2 (*CCND2*) [42-44] (Figure 2F). Taken together, our results confirm that E-box 1 in the *PIM3* locus is a functional target of Myc-mediated transcription in both human and mouse lymphoma cells.

### The Pim kinase inhibitor Pimi hampers cellular proliferation and causes a reduction in Myc regulated transcripts

**Figure 3:**
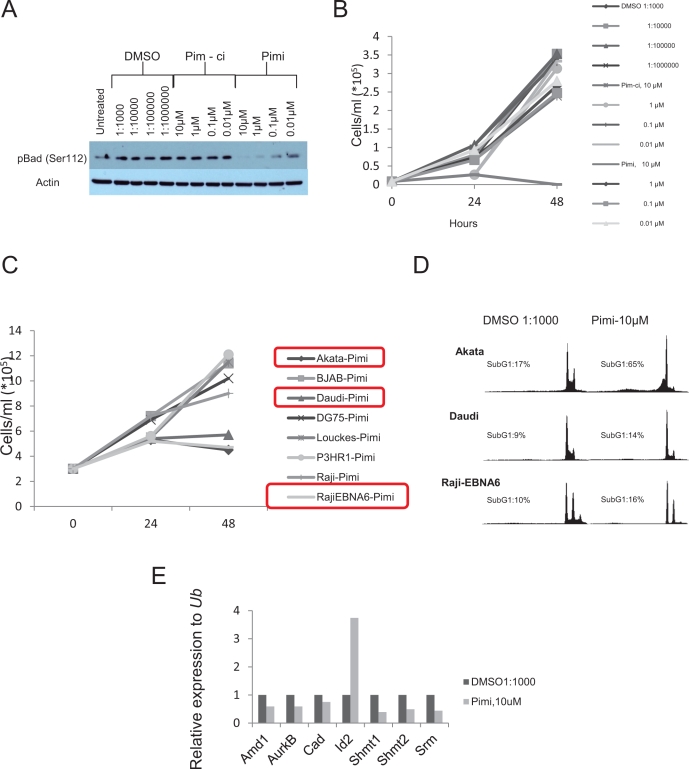
Inhibition of Pim kinases leads to de-phosphorylation of the pro-apoptotic protein Bad on serine 112 and induction of cell death (A) Eμ239 cells were cultured for 4 hours in the presence of the indicated concentrations of DMSO, Pim-ci or the Pim inhibitor Pimi, after which cells were harvested for western blot analysis of Bad serine 112 phosphorylation. B) Eμ239 cells were cultured in the presence of different concentrations of the indicated drugs and counted using trypan blue dye exclusion. C) Burkitt Lymphoma cell lines were cultured in the presence of 10 μM Pimi or DMSO (not shown) for 24 and 48 hours. At each time point aliquots of cells were taken for cell counting (by trypan blue dye exclusion) to monitor cell growth. D) At the indicated time points aliquots of cells were stained with propidium iodide and scored for the ratio of cells having less than a diploid DNA content (n<2), Sub G1, by FACS analysis. E) RNA from Eμ239 cells cultured for 24 hours in the presence of 10 μM Pimi was harvested and analyzed by qRT-PCR for expression of the Myc transcriptional target genes, *Amd1*, *Aurkb*, *Cad*, *Id2*, *Shmt1*, *Shmt2* and *Srm*. All values were normalized to that of the control primers, ubiquitin (*Ub*) run on the same sample.

Pim kinase expression has been associated with poor outcome in several different tumor types [45-58] and chemoresistance have been seen in tumor cells overexpressing the Pim kinases [59-62]. This has inspired efforts to inhibit the Pim kinases as a cancer treatment. Since the triple knockout *Pim* kinase mice are viable and fertile, this suggests that inhibition of the Pim kinase family could be possible without severe side effects [12]. To test this notion we obtained a specific pan-Pim kinase inhibitor (Compound 14j [63] [A953864.1] or from now on Pimi) generated by Abbott Laboratories (Abbott Park, IL). This inhibitor exhibits low nanomolar activity against Pim-3, Pim-1 and Pim-2 (0.5 nM, 2 nM, 3 nM, respectively) and shows high degree of selectivity for Pim kinases (personal communication, Dr. Joel Leverson, Abbott Laboratories). To investigate if this inhibitor hits the expected targets, Pim kinases, and exhibits anti-proliferative activity in Myc-induced lymphomas, we treated the mouse B-cell lymphoma cell lines Eμ239 and λ820 with Pimi and Pim-ci, a 1000-fold less potent Pim inhibitor from the same chemical series. As seen in Figure 3A, Western Blot analysis revealed that phosphorylation of the pro-apoptotic BH3-only Bcl-2 family member Bad on Ser112, which is believed to be a specific Pim kinase site, started to become decreased at 10 nM and was abolished in cells treated for 8 h with 10 μM Pimi, whereas Pim-ci did not affect phosphorylation. Moreover, measurement of incorporation of ^3^H-thymidine following 48 h of treatment with 10 nM to 10 μM Pimi demonstrated an EC50 of 2.6 μM or 6 μM for Eμ239 and λ820 cells, respectively (not shown). Since Pim-ci and the vehicle, DMSO, did not exert any anti-proliferative effects when added in the same relative quantities, we did not calculate EC50s for them.

To gain insight into the anti-proliferative effect of Pimi we generated growth curves on B cell lymphoma lines from mice and BL patients. Much like what was suggested from the ^3^H-thymidine incorporation assay, Eμ239 cells were sensitive to 10 μM Pimi, manifest as both slower growth and induction of cell death (Figure 3B). Surprisingly however, the anti-proliferative effect was only observed in a few human BL cell lines and growth curves and DNA histograms from FACS analysis of propidium iodide-labeled samples of the cell lines showed that Pimi was cytotoxic in Akata cells but only cytostatic in Daudi and Raji-EBNA6 cells (Figure 3C and 3D).

Pim-1 has been reported to facilitate transcriptional activation of 20% of Myc transcriptional targets through phosphorylation of Serine10 on histone H3 (H3S10) [28]. The phosphorylation is mediated by Pim-1 interacting with Myc in the transcriptional active Myc-Max dimer, thus recruiting Pim-1 to E-boxes of active transcription. Since inhibition of Myc signaling in Myc-driven tumors can lead to growth arrest, senescence and cell death we entertained the idea that the observed effect of Pimi could be mediated by inhibition of Myc function. We therefore treated the mouse B cell lymphoma cell line Eμ239 with 10 μM Pimi for 24 hours, harvested mRNA and analyzed it using qRT-PCR for the Myc regulated genes *Amd1* [39], *Aurkb* [64], *Cad* [65], *Shmt1*, *Shmt2* [66] and *Srm* [39]. Interestingly, several of the Myc-regulated target genes were down-regulated in Pimi-treated cells compared to DMSO controls (Figure 3E). On the other hand, expression of the disputed Myc target gene *Id2* [67, 68], was induced by Pimi.

### Pimi treatment induces Caspase-3 independent cell death

**Figure 4:**
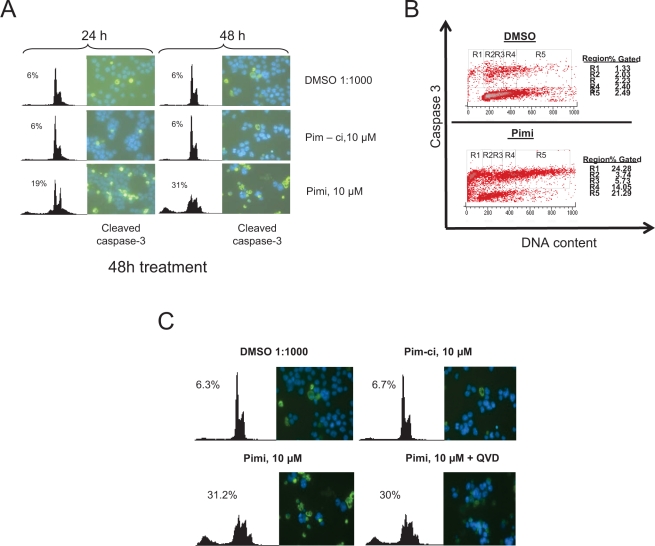
Pimi induces cleavage of caspase-3 in G2/M and aneuploid cells but cell death is not blocked by pan-caspase inhibition. A) Eμ239 cells cultured in the presence of DMSO, Pim-ci or Pimi was harvested at indicated timepoints and divided into two subsets. One set was stained with propidium iodide (PI) for FACS analysis and the other set was cytospun onto glass slides, labeled with cleaved caspase-3 antibody and a FITC-secondary antibody and then subjected to immunofluorescense analysis as described in materials and methods. B) Pimi treated Eμ239 cells were fixed and intracellularly labeled with cleaved caspase-3 antibody and 7-amino actinomycin D (7AAD). Cells were analyzed by FACS for quantification of the cleaved caspase-3 signal related to each phase of the cell cycle. C) Cells co-treated for 48 hours with either DMSO, Pim-ci or Pimi and 20 μM of the pan-caspase inhibitor QVD-OPH were harvested and stained with propidium iodide (PI) and analyzed by FACS as well as cytospun onto glass slides, labeled with cleaved caspase-3 and analyzed by immunofluorescence.

Phosphorylation of Bad by Akt or Pim kinases renders this protein inactive as an inducer of apoptotic cell death. Since Pimi blocked Bad phosphorylation and since we observed cells with a sub-G1 DNA content in Pimi-treated cells, we wanted to investigate if the cells died by classical caspase-dependent apoptosis. Immunofluorescense on cells treated with Pimi for 24 and 48 hours with antibodies directed against the cleaved form of the death effector caspase-3 showed marked increase in caspase-3 positive cells in Pimi-treated cells (Figure 4A). This was confirmed by FACS analysis on the same cells run in parallel. To define which phase of the cell cycle Pimi-treated cells died in, we performed a FACS analysis of fixed cells labeled with an antibody targeting cleaved caspase-3. Co-staining of DNA using 7-aminoactinomycin D (7-AAD) enabled us to analyze how caspase-3 positive cells were distributed in the cell cycle. As shown in Figure 4B, Pimi treatment led to caspase-3 activation in the G2/M phase (region 4) and in cells with hyperdiploid DNA content (>4n, region 5), suggesting that cells were undergoing death because of a problem in mitosis. To test whether Pimi-induced cell death is caspase-dependent, the pan-caspase inhibitor QVD-OPH was used together with Pimi. Immunofluorescense and FACS analyses showed that QVD-OPH reduces the intensity of caspase-3 staining but does not block Pimi-activated cell death (Figure 4C). These data indicate that inhibition of Pim kinases either results in caspase-independent cell death or that the caspase inhibition required to block death is not pharmacologically achievable.

## DISCUSSION

Here we show that the *Pim3* gene contains conserved E-boxes, one of which is bound by Myc, resulting in induction of transcription. This is the first time Myc has been implicated in regulation of a Pim kinase family member and it explains the high levels of Pim-3 found in Myc overexpressing mouse B cell lymphomas and human BL. It may also explain why *Pim1* and *Pim2* are more frequently trapped by proviral insertions of retroviruses in Myc-transgenic mice and why these two genes need to be deleted in order to see selection for *Pim3* trapping [9]. This in turn indicates that Pim-1 and Pim-2 kinases have non-redundant functions when expressed at normal levels but that they can be replaced by massive overexpression of Pim-3. Taken together these data indicate that the best rationale for developing Pim kinase inhibitors is to generate pan-inhibitors of all three family members, especially since triple knockout Pim kinase mice are viable.

Regulation of the Pim kinase family has focused mostly on Pim-1 and Pim-2, which are regulated by the Jak/STAT-pathway and by NfκB [6, 10, 69-75]. Significantly less is known about how Pim-3 expression is regulated, albeit one report shows that Pim-3 can be regulated by the Ets family of transcription factors in NIH 3T3 and human Ewing’s sarcoma cells [76]. Later studies showed that Pim-3 is activated by the Ets-1 transcription factor in pancreatic cancer cells [77]. Considering that Pim kinases are constitutively active and protein levels are mainly regulated through transcriptional activation and proteosomal degradation [21, 78], our finding that Pim-3 is regulated by Myc represents a significant addition to our understanding of Pim-3 regulation. It may also constitute an example of a feed-forward loop, since Pim-3 recently was shown to stabilize c-Myc [79]. This could offer an alternative explanation to why Myc’s transcriptional activity is decreased by Pimi-treatment in Eμ239 cells.

New targeted therapies are needed [80] and inhibition of Pim kinases has evoked a lot of interest but thus far no inhibitor has been granted approval for clinical use. Here we show that Myc-induced B cell lymphomas are sensitive to a new Pim kinase inhibitor generated at Abbott Laboratories, Pimi [63]. Whether or not Pim-3 is the sole target whose inhibition is the cause of the observed effect is not formally proven since RNAi against *Pim3* yielded incomplete and thereby inconclusive knockdown data (not shown). Nevertheless, Pimi induces cell death that correlates with but may be independent of caspase-3 activation. The reason why we cannot exclude caspase-dependent death is that there is a debate whether or not complete caspase inhibition is achievable pharmacologically [81]. Here we observe a considerable reduction in cleaved caspase-3 with QVD-OPH but the remaining amount may be sufficient to trigger death. If it is not classical caspase-dependent apoptosis then autophagy and/or programmed necrosis are candidate mechanisms. Indeed, autophagy was recently shown to be triggered by Pim-3 inhibition [79]. Given that Myc regulates metabolism [82] it would be plausible that interference with Myc’s transcriptional function would induce major changes in tumor cell metabolism that could result in apoptosis, autophagy and/or necrosis.

## MATERIALS AND METHODS

### Reagents

Primary antibodies were obtained from Cell Signaling (Pim-3 (D17C9), pBad (Ser112, 40A9), Myc (D84C12), p4E-BP1 (Thr37/46), and Caspase-3 (Asp175)), Santa Cruz (Pim-1(12H8), Pim-2(1D12), and Myc (N262)), Abcam (Pim-3) and Sigma (β-actin). Horseradish peroxidase-conjugated antibodies against mouse and rabbit antibodies were from GE Healthcare Life Sciences. The pan-caspase inhibitor Q-VD-OPH was obtained from Biovision. The Pim inhibitor, Pimi (2-Dimethylaminomethyl-8-(4-hydroxy-phenyl)-3H-benzo[4,5]thieno[3,2-d]pyrimidin-4-one), and the non-active control substance, Pim-ci, were obtained from Abbott Laboratories.

### Cell culture

NIH 3T3 fibroblasts were purchased from American Type Culture Collection and where cultured in Dulbecco's Modified Eagle Medium supplemented with 10 % fetal calf serum FCS, 2 mM L-Glutamine, 1 mM sodium pyruvate and 1 x antibiotic-antimycotic cocktail (penicillin/streptomycin/fungizone; Invitrogen). Human lymphoma cell lines P493-6 (a kind gift from Dr. Georg Bornkamm), human Burkitt Lymphoma cell lines; Akata, BJAB, Daudi, DG75, Louckes, P3HR1, Raji, Raji-EBNA6 and mouse lymphoma cell lines established from tumors arising in either λ*-Myc* or Eμ*-Myc* transgenic mice [29, 83] were cultivated in RPMI1640 medium supplemented with 10 % FCS, 2 mM L-glutamine, 50 μM β-mercaptoethanol, 0.1875 % sodium bicarbonate and 50 μg/ml gentamicin. For Myc-ER experiments cells were grown in the presence or absence of 4-hydroxytamoxifen (4-HT) and/or cycloheximide (both from Sigma) for 4 h followed by RNA harvest and analysis.

### Cell cycle and apoptosis analysis

For cell cycle analysis, suspension cells were harvested by centrifugation after which the pellet was dissolved in Vindelöv's solution (10 mM Tris, 10 mM NaCl, 75 μM propidium iodide, 0.1% Igepal and 700 units/liter RNase adjusted to pH 8.0) to a final cell concentration of 1*10^5^ − 1*10^6^ c/ml. The cells were then analyzed in a FACScalibur flow cytometer (BD Biosciences) plotting cell cycle distribution in linear mode in the FL3 channel on the x-axis. Apoptotic cells were analyzed as having less than a diploid genome and depicted as sub-G1 cells in a logarithmic FL-2 channel. For cleaved caspase-3 analysis, cells were fixed in 3% paraformaldehyde-PBS and permeabilized in ice-cold 90% methanol. Incubation with primary antibody against cleaved caspase-3 (Cell signaling) was performed overnight and was followed by incubation with a FITC-conjugated secondary antibody (Dako) for 1 h. Cells were then stained with 7-aminoactinomycin D (7AAD; Sigma) and analyzed by FACS analysis on a FACScalibur flow cytometer (BD Biosciences) using the FL1 (cleaved caspase-3) and FL2 (7-AAD) channels.

### Protein extraction and western blotting

Cells and harvested lymphomas were snapfrozen in liquid nitrogen and stored at −80 °C until further processing. Lymphomas were crushed in liquid nitrogen before addition of Tween-20 lysis buffer (50 mM Hepes, pH 7.5 /150 mM NaCl /1 mM EDTA/2.5 mM EGTA/0.1% Tween-20/ 1 mM phenylmethylsulfonyl fluoride/10mM β-glycerophosphate/1mM NaF/ 0.1mM NaVO4 supplemented with Minicomplete protease inhibitor cocktail tablets (Roche)) followed by sonication at 3 x 7sec pulses in a Soniprep 150 MSE, 20% power. Cell debris was pelleted by centrifugation at 1500 rpm (5 minutes, 4 °C) and supernatant was transferred to a fresh tube. Protein concentration was determined using the Bio-Rad protein determination kit. 50 μg of protein samples were separated on SDS-PAGE gels and transferred to nitrocellulose membranes (Schleicher-Schuell) activated in deionized water. Membranes were stained with Ponceau S Red dye to visualize equal loading and all subsequent steps were carried out in TBS-Tween (10 mM Tris-HCl, pH 7.6, 150 mM NaCl and 0.05% Tween-20) either containing 5% milk (blocking and antibody incubations) or 2 % BSA (phosphospecific antibody incubations). Antibody binding was visualized by enhanced chemiluminescence using the SuperSignal West Dura or Pico reagents from Pierce and either an X-ray film or a LAS4000 imaging system (Fujifilm Lifescience).

### RNA preparation and analysis

RNA was isolated using Trizol reagent (Invitrogen) or the NucleoSpin RNA II kit (Macherey-Nagel). Synthesis of cDNA was made with iScript first strand synthesis kit (Bio-Rad) on 1 μg RNA. Quantitative PCR (qPCR) was performed using the KAPA SYBR FAST qPCR Kit (Kapa Biosystems), cDNA and primers directed against *Pim1*, *Pim2*, *Pim3*, *Srm*, *Myc*, *Amd1*, *Aurkb*, *Cad*, *Shmt1*, *Shmt2* or *Ub* (Ubiquitin) on an iCycler iQ5 real-time PCR machine (Bio-Rad). Relative mRNA levels were calculated using the ΔΔCTmethod. Primer sequences are available on request.

### Chromatin immunoprecipitation (ChIP)

P493-6 cells were grown in the presence of 0.1 ug/ml tetracycline (Myc off) for 72 hours after which they were cleared of dead cells by centrifugation on a Ficoll (GE healthcare) gradient. One culture was continuously cultured in the presence of tetracycline (Myc off) whereas the other culture was grown in media only (Myc on). After 8 hours aliquots from each culture were taken for protein and mRNA analysis and the rest of cells (approximately 30 *10 ^6^ cells) where cross-linked using 1 % formaldehyde for 10 minutes. λ820 cells were expanded in normal media to correct cell density and were then cross-linked and handled as described before. The ChIP protocol was performed according to the manufacturer’s instructions (SimpleChIP enzymatic chromatin IP kit, Cell Signaling). Immunoprecipitations was performed using Myc antibodies (λ820 cells; D84C12, Cell Signaling, P493-6 cells; N262, SantaCruz). qPCR was run on immunoprecipitated and control chromatin using primers against E-boxes in the mouse and human Pim-3 gene. Control primers were designed against a region outside the *Pim3* gene. A 1:50 volume of chromatin was processed without antibody and used as total input control for quantitative PCR. Percentage total chromatin immunoprecipitated was calculated using the equation % total = 2 ^(Ct input - Ct ChIP)^ x % input used for chromatin immunoprecipitation (ChIP). As positive controls for Myc transcriptional regulation, primers directed against the established Myc target genes *Srm* and *Odc* were used in the ChIP assay on λ820 cells, whereas primers against *CCND2* were used for P493-6 cells. Sequences for primers are available on request.

### Immunofluorescence and immunohistochemistry

Following treatment cells were harvested by cytospin onto glass slides. The cells were fixed and permeabilized with a PBS solution containing 4% paraformaldehyde and 0.2% Triton X-100 for 20 minutes and then washed in PBS and blocking solution (5% FCS in PBS). After 1 hour of blocking, primary Caspase-3 antibody in blocking solution was added, and the slides were incubated overnight at 4°C in a moisture chamber. The cells were washed and incubated with a fluorescein isothiocyanate (FITC)–conjugated secondary antibody and subsequently stained with Hoechst and mounted. The slides were analyzed by fluorescence microscopy.

For immunohistochemistry, 2-μm sections of lymphoma paraffin blocks were deparaffinized. Antigen retrieval was performed by pressure cooking in citrate buffer (pH6) for 7min. Primary antibodies against Pim-3 (Abcam) were incubated overnight at 4°C. Antibody detection was performed using the DAKO REAL^TM^ detection kit (DAKO) according to the manufacturer’s protocol.

### [^3^H]-thymidine incorporation

Cells were cultured in 96-well plates for 48 h in the presence of either DMSO or various concentrations of Pimi. The last 2 h of incubation, 20 μCi of [^3^H]-thymidine (Amersham)/ml media was added and the cells were harvested by a semiautomatic cell harvester (Inotech CH-5605 Dottikon). [^3^H]-thymidine incorporation was measured using a liquid scintillation counter (PerkinElmer).
